# No increased inbreeding avoidance during the ovulatory phase of the menstrual cycle

**DOI:** 10.1017/ehs.2022.41

**Published:** 2022-09-28

**Authors:** Iris J. Holzleitner, Julie C. Driebe, Ruben C. Arslan, Amanda C. Hahn, Anthony J. Lee, Kieran J. O'Shea, Tanja M. Gerlach, Lars Penke, Benedict C. Jones, Lisa M. DeBruine

**Affiliations:** 1 Institute of Neuroscience and Psychology, University of Glasgow, Glasgow, UK; 2 School of Social Sciences, University of the West of England, Bristol, UK; 3 Institute of Psychology, University of Goettingen, Goettingen, Germany; 4 Max Planck Institute for Human Development, Berlin, Germany; 5 Department of Psychology, Cal Poly, Humboldt, CA, USA; 6 Division of Psychology, University of Stirling, Stirling, UK; 7 School of Psychological Sciences and Health, University of Strathclyde, University of Strathclyde, UK; 8 Leibniz-ScienceCampus ‘Primate Cognition’, Goettingen, Germany; 9 School of Psychology, Queen's University, Belfast, UK

**Keywords:** kinship, endocrinology, inbreeding avoidance, fertility, kin affiliation

## Abstract

Mate preferences and mating-related behaviours are hypothesised to change over the menstrual cycle to increase reproductive fitness. Recent large-scale studies suggest that previously reported hormone-linked behavioural changes are not robust. The proposal that women's preference for associating with male kin is down-regulated during the ovulatory (high-fertility) phase of the menstrual cycle to reduce inbreeding has not been tested in large samples. Consequently, we investigated the relationship between longitudinal changes in women's steroid hormone levels and their perceptions of faces experimentally manipulated to possess kinship cues (Study 1). Women viewed faces displaying kinship cues as more attractive and trustworthy, but this effect was not related to hormonal proxies of conception risk. Study 2 employed a daily diary approach and found no evidence that women spent less time with kin generally or with male kin specifically during the fertile phase of the menstrual cycle. Thus, neither study found evidence that inbreeding avoidance is up-regulated during the ovulatory phase of the menstrual cycle.

## Graphical abstract

**Figure:**
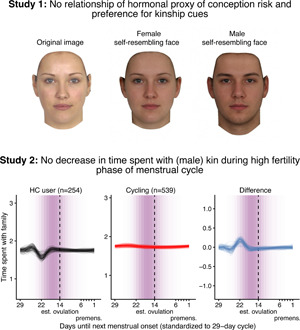

**Social media summary:** Neither preferences for facial kinship cues nor time spent with male kin decrease when women's fertility is high.

Many researchers have proposed that during the ovulatory (i.e., high-fertility) phase of the menstrual cycle, women's preferences for potential mates who will increase their reproductive fitness will strengthen or that women's aversions to potential mates who will decrease their reproductive fitness will strengthen (see Gildersleeve et al., 2014; Gangestad & Thornhill 2008; Jones et al., 2008 for reviews). Increased attraction to men displaying putative good-fitness cues (Gangestad et al., 2004, 2007; Penton-Voak et al., 1999; Penton-Voak & Perrett, 2000) during the ovulatory phase of the menstrual cycle are particularly high-profile (but not the only) examples of evidence that are widely cited for this claim.

Recently, however, the robustness of the evidence for ovulatory shifts in women's mate preferences has been called into question. For example, two different meta-analyses of this literature drew very different conclusions about the robustness of the evidence for ovulatory shifts in women's mate preferences (Gildersleeve et al., 2014; Wood et al., 2014). Researchers have also highlighted several potentially important methodological limitations of studies on this topic (Arslan et al., 2021; Blake et al., 2016; Gangestad et al., 2016; Jones et al., 2018a, 2019).

First, many researchers have emphasised that the majority of studies reporting significant ovulatory shifts in these behaviours are badly underpowered (Gangestad et al., 2016; Jones et al., 2018a). In combination with publication bias, this issue means that many of the published effects are likely to be false positives.

Second, many studies in this literature have employed between-subjects (i.e., cross-sectional) designs, which are ill-suited for testing subtle ovulatory shifts in behaviours that have substantial between-subject variance (Gangestad et al., 2016; Jones et al., 2018a). Importantly, large-scale within-subject (i.e., longitudinal) studies that used more objective methods to assess women's hormonal status (e.g., measuring sex hormones from saliva) have generally not replicated previously reported findings for ovulatory shifts in mate preferences (Jones et al., 2018a; Stern et al., 2020, 2021; Jünger et al., 2018; Marcinkowska et al., 2018).

Third, studies have typically used self-report methods to assess position in the menstrual cycle (e.g., self-reported number of days since last period of menstrual bleeding at time of testing). Empirical studies suggest these are imprecise and prone to bias (Blake et al., 2016), although this may not be a problem in longitudinal studies with very large samples (e.g., Arslan et al., 2021).

Since inbreeding bears several fitness-reducing costs, behaviours may have evolved to reduce the opportunities of inbreeding (Lieberman & Antfolk, 2016). These behaviours are predicted to especially increase around women's ovulation since this is the only time for women to get pregnant (Lieberman et al., 2011). However, these theories have yet to be subjected to large-scale, rigorous tests. To date, the best evidence for ovulatory shifts in inbreeding avoidance comes from Lieberman et al. (2011). In a longitudinal study of 48 women's mobile phone records from one menstrual cycle, Lieberman et al. reported that women called their fathers less frequently and spoke to them for less time when they did call them during the high-fertility phase of the menstrual cycle than when fertility was low. Because Lieberman et al. observed no such change in women's frequency or duration of calls to their mothers, they interpreted these results as evidence for adaptations that function to reduce opportunities for inbreeding to occur around ovulation. Consistent with Lieberman et al.'s findings, DeBruine et al. (2005) found that women showed stronger preferences for faces manipulated to possess kinship cues during the luteal (low-fertility) phase of the menstrual cycle than during the ovulatory phase in a cross-sectional study of 71 women. However, DeBruine et al. (2005) also found that preferences for cues of kinship in women's, but not men's, faces were related to women's progesterone level, but not estimated fertility. Both progesterone and fertility were estimated by converting reported menstrual cycle day to progesterone and conception risk values using actuarial tables.

Researchers have recently emphasised the importance of investigating cyclic shifts in behaviours that have not yet been the target of large-scale studies, including inbreeding avoidance (Jones et al., 2019). Thus, we revisited the claim of hormonal regulation of inbreeding-avoidance behaviours.

In Study 1, we examined this claim in a large-scale longitudinal study of the relationship between women's (*N* = 199) salivary hormone levels and their responses to kinship cues in faces. Following previous studies of responses to facial kinship cues (DeBruine, 2002, 2004, 2005; DeBruine et al., 2005), we experimentally manipulated male and female face images to be more or less similar in shape to our participants’ faces and assessed the effects of this manipulation on perceptions of attractiveness and trustworthiness. Previous research has shown that this image manipulation can reliably tap inbreeding-avoidance behaviours. For example, women show aversions to opposite-sex faces with similar shape characteristics to their own when assessing men for exclusively sexual relationships, such as one-night stands, but not when assessing their trustworthiness (DeBruine, 2005). Moreover, such effects are not due to feminisation of opposite-sex faces when increasing self-resemblance to female participants (DeBruine, 2005). Further evidence that people respond to this image manipulation in ways consistent with it functioning as a kinship cue comes from studies showing that people are more likely to cooperate with people with similar face-shape characteristics (DeBruine, 2002) and perceive them to be more trustworthy (DeBruine, 2005).

The ovulatory phase of the menstrual cycle is characterised by the combination of high estradiol and low progesterone (Gangestad & Haselton, 2015). Thus, if Lieberman et al. (2011) are correct that ovulation increases inbreeding-avoidance behaviours, we would expect preferences for self-resembling male, but not self-resembling female, faces to decrease when estradiol is high, and progesterone is simultaneously low.

In Study 2, we tested for hormonal regulation of inbreeding-avoidance behaviours in a more ecologically valid study, namely a daily diary study. Similar to Lieberman et al. (2011), we investigated whether women spent more time with their families and had more contact with male kin during the fertile phase. Because we assume phone calls to be a less valid proxy, we assessed the frequency of actual contact as well as thoughts about male kin across women's ovulatory cycle. Further, inbreeding avoidance should not be limited to father–daughter relationships. Therefore, we investigated contact to all male kin across the ovulatory cycle.

## Study 1

Study 1 investigated whether women's responses to kinship cues in faces track changes in estradiol and progesterone.

### Methods

#### Participants

Women participated as part of a large study of possible effects of steroid hormones on women's behaviour (Jones et al., 2018a–c). The total sample size was determined by resource availability and the exclusion criteria that were part of the associated original grant proposal. For the current study, we report data from all 205 heterosexual women (mean age = 21.5, SD = 3.3 years) from blocks of test sessions where women were not using any form of hormonal contraceptive (i.e., reported having a natural menstrual cycle) and completed the face-judgement task in at least two test sessions. Participants completed up to three blocks, each of which consisted of five weekly test sessions. One hundred and seventy-two women had completed four or more test sessions and 41 of these women completed nine test sessions. Thirty-three women completed fewer than five test sessions. Six of these women only had valid hormone data for one session and were excluded; the remaining 199 women were included in the longitudinal analyses below.

#### Procedure

**Figure 1.**
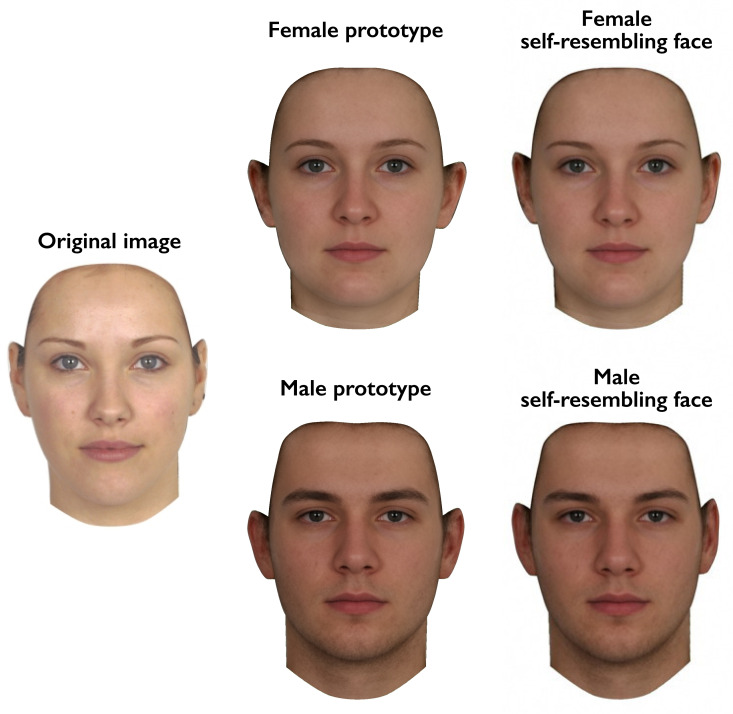
Self-resembling stimulus faces were created by applying 50% of the difference in shape between an individual's face and the female prototype to both female and male prototype faces.

In the first test session, a full-face photograph of each woman was taken under standardised photographic conditions. Camera-to-head distance was held constant. These photographs were used to manufacture self-resembling faces using the same methods as previous research (DeBruine, 2002, 2004, 2005; DeBruine et al., 2005). Self-resembling faces were created by applying 50% of the shape difference between each participant's face and a same-sex (i.e. female) prototype face to same-sex and opposite-sex prototypes, to produce same-sex and opposite-sex self-resembling faces. Importantly, this method for manipulating self-resemblance in opposite-sex faces (DeBruine, 2004) avoids the feminisation of male stimulus faces that occurs when simply blending self and opposite-sex faces. Male and female comparison stimuli that resembled none of the participants were manufactured in the same way using images of 10 women who did not participate in the study. As in previous research on responses to self-resembling faces (DeBruine, 2002, 2004), image manipulations were carried out using specialist computer graphic software (DeBruine, 2018; Tiddeman et al., 2001). Example stimuli are shown in Figure 1.

In all subsequent test sessions (all test sessions after the first), each woman completed a face-judgement task in which they were presented with 20 pairs of faces. Ten of these pairs consisted of a self-resembling face and a comparison face. The other 10 pairs consisted of a non-resembling face (constructed from another randomly selected age-matched woman participating in the study) and the same comparison faces. This method allows us to compare judgements of self-resembling faces with judgements of non-resembling faces, while keeping equal the number of times self- and non-resembling faces are presented.

Participants were instructed to click on the face in each pair that they thought looked more attractive or, in a separate block of trials, more trustworthy. Trial order and the side of the screen on which any given image was presented were fully randomised. In each test session, each woman completed the face-judgement task four times. In the first version, they were presented with female faces and judged attractiveness. In the second version, they were presented with female faces and judged trustworthiness. In the third version, they were presented with male faces and judged attractiveness. In the fourth version, they were presented with male faces and judged trustworthiness. The order in which participants completed these versions of the face-judgement task was fully randomised.

#### Saliva samples

Participants also provided a saliva sample via passive drool (Papacosta & Nassis, 2011) in each test session. Participants were instructed to avoid consuming alcohol and coffee in the 12 hours prior to participation and avoid eating, smoking, drinking, chewing gum or brushing their teeth in the 60 minutes prior to participation. Each woman's test sessions took place at approximately the same time of day to minimise effects of diurnal changes in hormone levels (Veldhuis et al., 1988; Bao et al., 2003).

Saliva samples were frozen immediately and stored at −32°C until being shipped, on dry ice, to the Salimetrics Lab (Suffolk, UK) for analysis, where they were assayed using the Salivary 17β-Estradiol Enzyme Immunoassay Kit 1-3702 (*M* = 2.82 pg/mL, SD = 1.03 pg/mL, sensitivity = 0.1 pg/mL, intra-assay CV = 7.13%, inter-assay CV = 7.45%) and Salivary Progesterone Enzyme Immunoassay Kit 1-1502 (*M* = 157.2 pg/mL, SD = 104.9 pg/mL, sensitivity = 5 pg/mL, intra-assay CV = 6.20%, inter-assay CV = 7.55%). Hormone levels more than three standard deviations from the sample mean for that hormone or where Salimetrics indicated levels were outside the sensitivity range of their relevant ELISA were excluded from the dataset (~0.1% of hormone measures were excluded for these reasons). The descriptive statistics given above do not include these excluded values and do not include statistics for the first test session where women did not complete the face-judgement task. Values for each hormone were centred on their subject-specific means to isolate effects of within-subject changes in hormones and were scaled so the majority of the distribution for each hormone varied from −0.5 to 0.5. This was done simply to facilitate calculations in the linear mixed models. Since hormone levels were centred on their subject-specific means, women with only one value for a hormone could not be included in these analyses.

### Analyses and results

Analyses were conducted using R version 4.1.2 (R Core Team, 2018). We fitted Bayesian mixed-effects models implemented in Stan (Carpenter et al., 2017) via the brms package (version 2.15.0; Bürkner, 2017, 2018). Random slopes were specified maximally following Barr et al. (2013) and Barr (2013). Models were specified with brms-default flat priors for the population-level effects and default weakly informative priors for the variance components. All our models converged as suggested by Rhat (Gelman & Rubin, 1992). Data, full results and code for all analyses, as well as model checks are publicly available at https://osf.io/wnhma/.

Face sex was effect-coded (−0.5 = female, +0.5 = male), as was judgement type (−0.5 = attractiveness, +0.5 = trustworthiness) and stimulus type (−0.5 = control-resembling, +0.5 = self-resembling). The dependent variable was whether in any given trial the target face (1) or comparison face (0) was chosen. Note that women with only a single test session where they completed the face-judgement task and had valid estradiol and progesterone levels cannot be included in these longitudinal analyses (*n* = 6). Thus, data from 199 women were included in these analyses.

#### Model 1: E, P and E-to-P ratio

**Figure 2.**
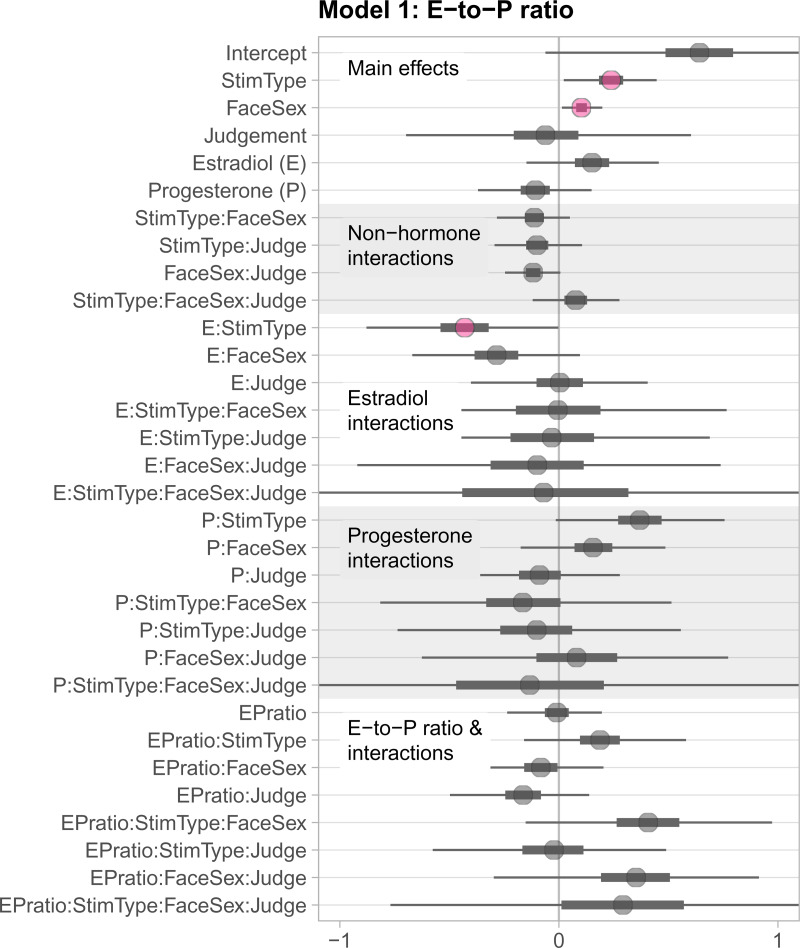
Treeplot summarising all estimates from Model 1. Plotted uncertainty intervals are 99% CIs. The inbreeding avoidance hypothesis would predict that when estradiol is high and progesterone low (i.e. *EPratio* high), self-resembling (*stim type*) male faces (*face sex*) would be less preferred than control-resembling male faces, and particularly so for ratings of attractiveness compared with ratings of trustworthiness (*judgement type*). However, credible intervals for both the interaction of *EPratio × stimulus type × face sex × judgement type* and the lower-order interactions without judgement type included 0. The weakest form of the inbreeding avoidance hypothesis would predict a simple interaction of fertility (*EPratio*) and avoidance of self-resembling faces (*stimulus type*), independent of face sex and judgement type; again, we found no evidence for such an interaction.

In the first model (Model 1) we included estradiol (scaled and centred), progesterone (scaled and centred), estradiol-to-progesterone ratio (E-to-P ratio; scaled and centred), face sex, judgement type and stimulus type as predictors, as well as all possible interactions among these predictors except interactions including both estradiol and progesterone (which is represented by the E-to-P ratio). A summary of all estimates and their associated 99% credible intervals (CI) is shown in Figure 2. While 89% CIs are considered more stable than wider CIs (e.g., Kruschke, 2014), we reported 99% CIs for consistency with the preregistered analyses from Study 2. A table of all estimates can be found in the Supporting Information at https://osf.io/wnhma/.

#### Interaction(s) of interest

DeBruine et al. (2011) reported interactions among judgement type, face sex and self-resemblance. Therefore, we predicted to find a four-way interaction of judgement type, face sex, stimulus type and estradiol-to-progesterone ratio: for judgements of male (but not necessarily female) attractiveness (but not necessarily trustworthiness), the E-to-P ratio (as a proxy for female fecundity) should be associated with an avoidance of target faces in the self-, but not control-resembling trials. However, patterns of results with lower-order interactions that include self-resemblance and E-to-P ratio could also inform the incest avoidance hypothesis if judgements of male attractiveness follow the predicted pattern of being negatively linked to conception risk proxies.

Contrary to our prediction, the credible interval for the predicted four-way interaction included 0 (estimate = 0.29, 99% CI [−0.77, 1.33]), indicating that the four predictors did not interact to predict face preferences. 99% CIs for relevant lower-order interactions also included 0 (see Figure 2).

##### Additional focused analyses

To directly assess only the main effect of interest, we also conducted a targeted analysis of only male attractiveness judgements. As for the full model, we entered estradiol (scaled and centred), progesterone (scaled and centred), E-to-P ratio (scaled and centred) and stimulus type as predictors, as well as all possible interactions among these predictors. Other than for the intercept (estimate = 0.72, 99% CI [0.02, 1.42]), the CIs for all estimates included 0. Specifying weakly informative priors (see Supporting Information) led to all 99% CIs including 0.

Finally, we ran a last model that included only E-to-P ratio, stimulus type and their interaction as predictors. All CIs included 0.

#### Other effects

The 99% CIs for several other effects that were not relevant to the main hypothesis did not include 0. These effects are summarised here and detailed with visualisations for all interactions in the Supporting Information.

The estimate for the main effect of stimulus type was 0.24 (99% CI [0.02, 0.45]), indicating that self-resembling target faces were more likely to be chosen compared with control-resembling faces. The main effect of stimulus type was also qualified by an interaction with estradiol (estimate = −0.43, 99% CI [−0.88, −0.00]). Estradiol showed a positive association with a preference for control-resembling (but not self-resembling) faces; at higher levels of estradiol, control-resembling faces were chosen more often. The estimate for the main effect of face sex was 0.10 (99% CI [0.01, 0.20]) indicating that male target faces were more likely to be chosen than female target faces.

### Model 2: E, P and E × P interaction

**Figure 3.**
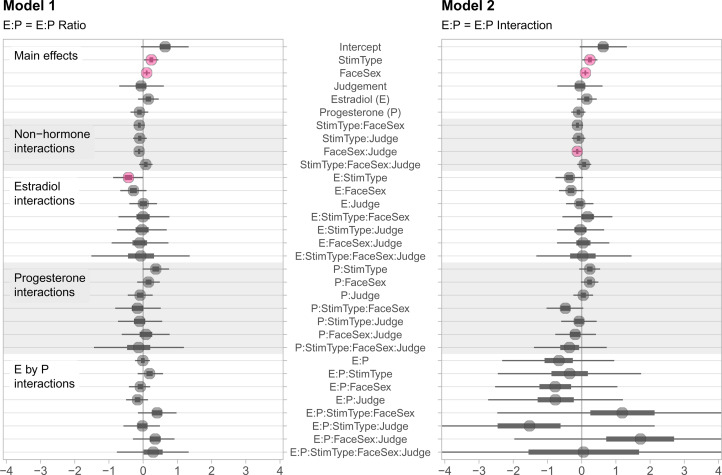
Treeplot summarising all estimates from Models 1 (E-to-P ratio, left) and 2 (E ×P interaction, right). Plotted uncertainty intervals are 99% CIs.

The second model (Model 2) that we tested included estradiol (scaled and centred), progesterone (scaled and centred), face sex, judgement type and stimulus type as predictors, as well as all possible interactions among these predictors. The role of the estradiol-to-progesterone ratio in Model 1 is now represented by the interaction between estradiol and progesterone. A summary of all predictors and their associated 99% credible intervals is shown in Figure 3. A table of all parameter estimates can be found in the Supporting Information at https://osf.io/wnhma/.

#### Interaction of interest

Based on the inbreeding avoidance hypothesis, we predicted finding a five-way interaction of judgement type, face sex, estradiol, progesterone and stimulus type: for judgements of male attractiveness, E and P should interact in their association with face preferences, so that when E is high and P is low, male self-resembling faces should be less preferred than male control-resembling faces.

Contrary to our prediction, the credible interval for the predicted interaction included 0 (estimate = 0.06, 99% CI [−5.92, 6.23]), indicating that this interaction had no effect on face preferences, although the credible interval was very wide. As in Model 1, we found main effects for face sex and stimulus type. As opposed to Model 1, the 99% CI for the interaction of estradiol and stimulus type included 0, while the 99% CI for the interaction between face sex and judgement type did not include 0 (estimate = −0.13, 99% CI [−0.26, −0.00]). The main effect of face sex (i.e., male target faces were more likely to be chosen than female target faces) was qualified by an interaction with judgement type such that this effect was weaker for trust compared with attractiveness judgements.

All other effects observed in Model 2 (as well as additional focused analyses of the main effect of interest) followed the same patterns as those observed in Model 1 (see Supporting Information).

### Robustness check

We had initially conducted different analyses that can be found on the OSF (https://osf.io/wnhma/), using models identical to those we have used previously to test for hormonal regulation of women's masculinity preferences (Jones et al., 2018a), disgust sensitivity (Jones et al., 2018b) and sexual desire (Jones et al., 2018c).

Instead of using unaggregated responses on the 2AFC-task, we computed a self-resemblance preference score for each version of the face-judgement task, and then used linear mixed effect models in lme4 (version 1.1-18-1; Bates et al., 2015) to test for possible effects of hormonal status. The self-resemblance bias score was generated by subtracting the number of times the control-resembling faces were chosen (out of 10) from the number of times the self-resembling faces were chosen (out of 10). These four scores were calculated separately for each participant in each test session. Positive scores indicated a bias towards self-resembling (vs. control-resembling) faces, so higher scores indicated that self-resembling faces were perceived as more attractive or trustworthy than control faces. These models did not provide evidence in line with the inbreeding avoidance either, and can be considered robustness checks for the analyses reported here.

## Study 2

While Study 1 tested the association of directly measured hormone levels and preferences for experimentally manipulated kinship cues, Study 2 used a more ecologically valid outcome measure of self-reported behaviour towards actual kin, investigating whether women reported seeking more contact with their families and male kin during the fertile phase.

### Methods

#### Participants

In a preregistered online diary study on conception risk and women's mate preferences (Arslan et al., 2020b; see also https://osf.io/d3avf/), we were able to recruit a sample of 1,660 participants of whom 794 women fulfilled our inclusion criteria. The recruitment took place from June 2016 to January 2017 through a variety of channels (e.g., online platform psytests.de, advertisement on okCupid.com and Facebook and mass mailing lists of university students) as well as direct invitations of suitable candidates taking part in previous lab studies. Budget determined our sample size, with the incentives for taking part in the study being either direct payment of participants with an amount ranging from 25€ up to 45€ depending on their regularity of participation or the chances of winning in a lottery with prizes totalling 2000€. Students of the University of Goettingen were also able to earn course credit, with the total credit again depending on the regularity of their participation. At the end of the study, every participant received personalised graphical feedback as a further incentive.

We divided our sample into 539 (68%) women not taking hormonal contraceptives (mean age = 26.5, SD = 5.9 years) vs. a quasi-control group of 255 (32%) women who were taking hormonal contraceptives (mean age = 24.0, SD = 4.6 years). Women in our quasi-control group were older, had a longer self-reported cycle length, more previous sex partners and were less conscientious compared with women not taking any form of hormonal contraception (Table 1). Inclusion criteria for taking part in this study were being heterosexual and premenopausal, having a regular menstrual cycle, not taking steroid-based medication, being younger than 50 years of age, and neither breastfeeding nor being pregnant during the study or in the three months prior to participation. Women who could not be clearly classified as hormonal contraceptive users or non-users were also excluded (e.g., if they changed their contraceptive method during the course of the study or when they had gone off hormonal contraception less than three months prior to the study). Women who lived with their parents were also excluded because their living arrangements will have directly influenced contact with male kin. In our analyses on women's time spent with their families, we did not differentiate between male and female relatives (although women frequently met both male and female relatives on the same day). In our analyses of women's contact with related men, only single women were included, because only they answered detailed questions about who they spent their day with (women in a relationship answered questions about their partner instead). A total of 263 (33.12%) women were currently single with 203 women not using hormonal contraceptives (mean age = 24.4, SD = 4.9 years) and 60 women using hormonal contraceptives (mean age = 24.5, SD = 5.3 years). Exclusion criteria were relaxed and strengthened in our robustness analyses. We list all deviations to our preregistration in S1.

**Table 1.** Descriptive statistics by hormonal contraceptive use

Hormonal contraceptive use	No (*n* = 539)	Yes (*n* = 255)		
Variable	Mean (SD)	Mean (SD)	Hedges’ *g*	*p*
**Age**	**26.53 (5.85)**	**23.97 (4.58)**	**−0.44**	**<.001**
Religiosity	2.21 (1.33)	2.16 (1.34)	−0.04	= .616
Age at first sex	17.06 (2.83)	16.75 (2.65)	−0.11	= .142
Age at menarche	12.76 (1.38)	12.75 (1.26)	−0.01	= .906
**Cycle length (days)**	**28.99 (3.15)**	**27.64 (2.31)**	**−0.43**	**<.001**
**Lifetime sex partners**	**8.95 (11.00)**	**6.20 (9.14)**	**−0.25**	**<.001**
Extraversion	3.42 (0.76)	3.49 (0.81)	0.09	= .240
Agreeableness	3.67 (0.59)	3.74 (0.62)	0.12	= .127
Neuroticism	2.98 (0.76)	2.93 (0.77)	−0.06	= .404
**Conscientiousness**	**3.49 (0.65)**	**3.63 (0.69)**	**0.22**	**= .005**
Openness	3.82 (0.62)	3.73 (0.61)	−0.15	= .053

*Note:* All ages in years; religiosity assessed on a scale from 1 = not religious to 6 = religious; cycle length = self-reported average cycle length. Bold values indicate effects for which there was a significant difference between women not taking hormonal contraceptives and the quasi-control group of women taking hormonal contraceptives.

#### Procedure

Women participated in an online study named ‘Everyday life and sexuality’ implemented using the survey framework formr.org (Arslan et al., 2020a) with the aim of examining the interplay of sexuality, psychological well-being and romantic relationships with everyday experiences. Initial questionnaires that were administered at the start of the project assessed factors such as relationship status and hormonal contraceptive use. A day after these surveys, women started the online diary. Daily invitations were sent at 5 p.m. via email (or in the case of repeated non-response, text message) and questionnaires could be filled out until 3 a.m. the following day. After 70 days, the daily questionnaires ended. In the daily questionnaires, among other questions, women reported how they spent their time, as well as names or identifiers of people with whom they had social contact (‘With these people I had longer social contact (longer than 1 hour)’) and had thought about (‘I thought about these people a lot and would have liked to see them’). Every day, women could indicate whether their responses for a test session were dishonest or random, and such responses were discarded (fewer than 0.4% of days). In every third test session, women were asked to report the number of days since the onset of the last period of menstrual bleeding. For women who did not experience a menses onset in the last five days of the diary, the next menstrual onset was additionally assessed in a follow-up questionnaire. After finishing the daily diary, women completed a social network questionnaire that assessed their relationship to the individuals listed in the diary. The full procedure (including variables not analysed here) can be found on the OSF (https://osf.io/3fbgk/).

#### Fertility estimation

We defined the premenstrual phase as the six days preceding the next menstrual onset. Because we only asked about menstruation every three days and did not ask for the date of the end of the menstrual period, we imputed the probability of menstruation on days where it had not been measured.

The probability of being in the fertile window of the menstrual cycle was imputed for each test session using the backwards counting method following Gangestad et al. (2016) and Arslan et al. (2021). Women using hormonal contraception were also assigned a probability of being in the fertile window to serve as a quasi-control group, among whom the probability is unrelated to ovulation but still correlated with time since menses and other potential confounds. To graph the outcomes as a continuous function over cycle days, we standardised each cycle to a length of 29 days by ‘squishing’ the follicular phase to the same length for all participants. The luteal phase was not ‘squished‘ because it is less variable in length. Standardising cycle length has the benefit that the assumed cyclic cubic function we used connects the start and end of the cycle for participants with different cycle lengths.

#### Outcome measures

Our two main outcome measures were a Likert item assessing time spent with family (‘I sought contact with my family’ rated on a five-point scale from less often than usual to more often than usual) and a count variable for related men. The latter variable was formed by counting the number of contacts listed on that day in the diary that had been confirmed to be male relatives in the follow-up social network survey. Because our method of assessing identifiers first and then finding out more about each in a follow-up survey was experimental, several problems occurred. One, not all women completed the follow-up survey. Two, we asked women to list contacts longer than one hour, so we did not count contacts shorter than one hour (in a robustness check, we additionally included male kin that women reported thinking about/wanting to meet). Three, we intended to let women rate the 10 individuals whom they had most often listed in the diary. However, owing to a programming error, up to 10 social network members were instead rated in increasing frequency of contact. For contacts that were not rated, we could only infer the identity of the contact when women used clear labels (e.g., ‘Dad’, ‘Brother’), which we did as a robustness analysis. Finally, only single women listed their contacts each day. We made this design choice to equalise the number of survey questions between single and non-single women, but we recruited a smaller percentage of single women than we expected. As it turned out, the calculated incidence of contacts with related men was low (0.08 per day), many participants reported no confirmed contacts during the diary and the overall sample size was smaller than planned. However, we were able to use a larger portion of our sample for the Likert item and we could use the social network survey to confirm that many meetings with family included both male and female kin and that time spent with family was a proxy for number of male kin contacts (*r*_(# male kin, # female kin)_ = .40, *r*_(# male kin, time family)_ = .21). Time spent with family was positively correlated with the reported number of male kin contacts, r_(# male kin, time family)_ = .21). Although this correlation may seem low, it is attenuated by criterion invalidity in the social network measure. The correlation between the Likert item and the unobservable time spent with male kin should be substantially higher (see Supplementary Note S2).

#### Analyses

In our preregistration, we had predicted that women would spend less time with related men, and less time with family in general when fertile (i.e., an association with the fertile window probability only among women not using hormonal contraception). As preregistered, we tested this hypothesis by fitting Bayesian regression models implemented in Stan (Carpenter et al., 2017) via the brms package (Bürkner, 2017; Bürkner, 2018) with brms-default flat priors for the population-level effects and default weakly informative priors for the variance components. We preregistered multilevel models with varying intercepts and varying slopes for the fertile window probability at the participant level (see https://osf.io/d3avf/). We adjusted for current menstruation and premenstrual phase. Deviating from our preregistration, hormonal contraception was included as a potential moderator of all cycle phase predictors (see Table S1). In Wilkinson notation (Wilkinson & Rogers, 1973), our main model was specified as follows:
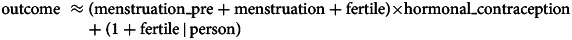


We assumed a normal distribution for regressions on the ‘time with family’ item and a Poisson distribution for the ‘number of related men seen’ on that day.

### Results and discussion

**Figure 4.**
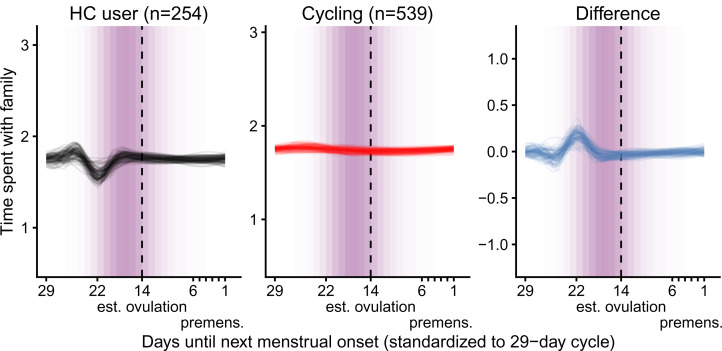
Change in time spent with family over the menstrual cycle. The *y*-axis range shows the mean ± 1SD (possible responses were 0–4).

**Figure 5.**
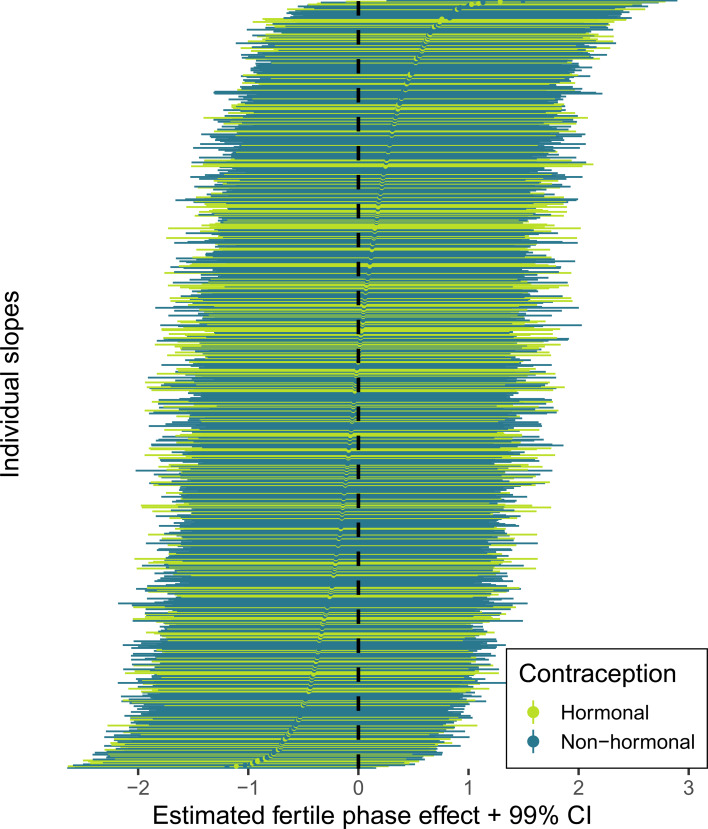
Forest plot of varying slopes of the fertile phase effect on time spent with family. Each line and dot represent the estimate and 99% credible intervals (CI) for the fertile phase effect on time spent with family, ordered by strength of the fertile phase change.

According to our preregistered decision criterion of *ɑ* = 0.01 (one-sided), we detected no fertile window decreases in contact with related men, nor in time spent with family (Table 2). Visually graphing a cyclic cubic spline over the days until the days of the next menstrual onset showed no dip in the fertile window for either outcome (see Figure 4, Figure S1). Women using hormonal contraceptives did not have more midcycle contact with male kin than women not using hormonal contraceptives, but rather had less. We found evidence of small but credible increases in the number of male kin seen during menstruation and the premenstrual phase. We found a similar increase during menstruation for time spent with family. These increases were of similar size for women on and off hormonal contraception. In the continuous graph, the most prominent feature is a slight post-menstrual dip among hormonal contraceptive users (Figure 4). We also examined contact with related women, unrelated men and unrelated women to test specificity and detected no changes during the fertile window either (Table S2). Neither did we observe fertile window decreases according to our criterion when counting the number of male kin the women thought about and would have liked to meet or when we lumped the number of male kin they actually met and only thought about and would have liked to meet (see Figure S2). Descriptively, the fertile window effect was in the predicted direction for both outcomes (but very close to zero for the Likert item). However, the direction of the effect was inconsistent across our robustness analyses. Figure 5 shows the individual slopes of the fertile window from the multilevel model on time spent with family. Notably, the estimated direction of the effect was inconsistent across participants. Several participants exhibited an increase in time spent with family during the fertile window.

**Table 2.** Model summary of the main models

	‘I sought contact with my family’		Number of male kin met >1 h	
Predictors	Estimates	CI (99%)	Incidence rate ratios	CI (99%)
Intercept	1.71	1.64; 1.78	0.00	0.00; 0.00
Premenstrual phase	−0.01	−0.08; 0.05	1.42	1.04; 1.95
Menstruation	0.08	0.01; 0.16	1.49	1.04; 2.09
Fertile phase	−0.01	−0.17; 0.15	0.65	0.01; 15.94
Hormonal contraception (HC)	0.06	−0.07; 0.20	0.64	0.05; 5.30
Premenstrual phase × HC	0.06	−0.07; 0.18	1.12	0.39; 3.54
Menstruation × HC	0.00	−0.15; 0.16	1.10	0.27; 4.26
Fertile phase × HC	0.08	−0.24; 0.37	0.18	0.00; 9.31
Group-varying effects				
*sd*(Intercept)	0.49	0.45; 0.54	44.25	16.78; 189.88
*sd*(Fertile phase)	0.69	0.54; 0.84	9.85	3.12; 100.81
*sd*(Residual)	1.13	1.11; 1.14	Not applicable	
*N* women	793		263	
*N* days	24 450		12 251	

*Note*: CI, credible interval; *sd*, standard deviation of varying effect.

All our models converged as suggested by Rhat (Gelman & Rubin, 1992). Full results and analysis code for all analyses, as well as additional robustness checks, can be found at https://osf.io/f2hct/).

#### Robustness checks

**Figure 6.**
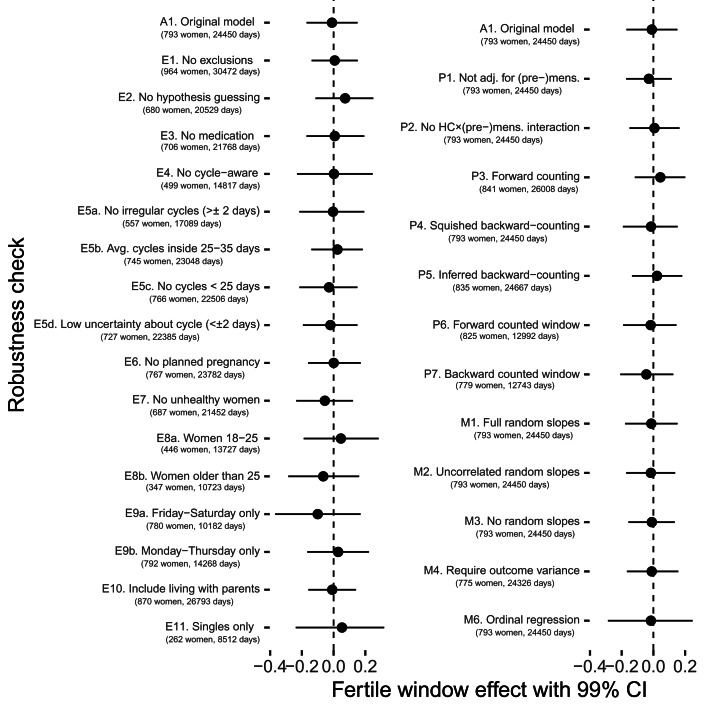
Robustness checks for fertile window changes in time spent with family.

In a series of preregistered robustness checks, we tested whether different exclusion criteria, fertile window estimation methods, modelling choices, and outcome definitions would have changed our results. Although effect sizes sometimes went descriptively in a different direction, 99% confidence intervals always included zero (with one exception in the opposite direction of the prediction) and in none of these analyses we would have found support for our hypotheses according to our preregistered criterion (see Figure 6 and Figure S2). As alternative exclusion criteria, we tested (1) no exclusions other than those necessary for estimating fertility, (2) additionally excluding women who approximately guessed that the study was about fertile window changes, (3) excluding women who reported using any psychopharmacological, hormonal or antibiotic medication, (4) excluding cycle-aware women, (5a) excluding women who reported cycles with more than 2 days variability in length, (5b) excluding women who reported average cycle lengths shorter than 25 or longer than 35 days, (5c) excluding cycles shorter than 25 days in the diary, (5d) excluding women who were uncertain about the length and regularity of their menstrual cycle, (6) women who were not trying to avoid pregnancy, (7) excluding women who reported feeling unhealthy, (8a) only women aged 18–25, (8b) only women 26 and older, (9a) only including Fridays to Sundays, (9b) only including Mondays to Thursdays, (10) including women who lived with their parents and (11) for the Likert item on time spent with family, excluding women in a relationship (for comparability with the male kin data subset). As alternative method to estimate the fertile window probability, we tested (1) not adjusting for (pre-)menstruation, (2) not adjusting for the interaction between hormonal contraception and (pre-)menstruation (as we had preregistered but now consider suboptimal), (3) using forward-counting from the last menstrual onset, (4) ‘squishing’ the follicular phase to a standard length before estimating fertile window probability, (5) counting backwards from the next menstrual onset inferred from the reported average cycle length, (6) using a discrete, rather than continuous fertile window predictor when forward counting and (7) using a discrete predictor when backward counting. With regard to other modelling choices, we (1) added varying slopes for the menstruation and premenstruation predictors, (2) added varying slopes but assumed them to be uncorrelated, (3) omitted varying slopes for the fertile window predictor, (4) required that the outcomes have variance for each participant, (5) assumed a normal distribution for the number of male kin and (6) estimated an ordinal regression rather than a Gaussian regression for time with family. Finally, we explored whether a different definition of the ‘number of male kin’ variable led to change by (1) adding male kin inferred by us from the identifier, but not explicitly rated in the social network follow-up, (2) counting male kin that women reported thinking about and wanting to meet and (3) counting male kin about whom women reported either physical contact or thoughts.

## General discussion

Neither study found clear evidence for effects in the direction expected if women actively avoid inbreeding during the fertile phase of the menstrual cycle. In Study 1, women's preference for self-resembling male faces was not related to estradiol-to-progesterone ratio or the interaction between estradiol and progesterone (common proxy measures of conception risk). In Study 2, women descriptively reported seeking less contact with family and meeting fewer male kin when they were in the fertile phase of their cycle according to backward-counting (another validated proxy measure of conception risk), but estimates did not meet our preregistered criterion and were directionally inconsistent across robustness checks and participants.

In Study 1, we tested for evidence of hormonally regulated inbreeding avoidance in a longitudinal study of women's responses to faces possessing kinship cues (i.e., self-resembling faces). In contrast with our predictions, we found no evidence that judgements of the attractiveness of male faces showed a self-resemblance aversion when fertility was high. Thus, our data from Study 1 do not support previous suggestions that inbreeding avoidance increases with conception risk during the menstrual cycle (DeBruine et al., 2005; Lieberman et al., 2011).

Overall, women did judge self-resembling faces, independent of face sex or hormone levels, to be more trustworthy and attractive than control-resembling faces. This replicates results from previous research showing self-resemblance preferences (DeBruine, 2002, 2004, 2005).

In a cross-sectional study, DeBruine et al. (2005) reported that self-resemblance preference increased when progesterone levels were relatively high when assessing female, but not male, faces. Evidence for such an effect in our sample was not present. DeBruine et al. (2005) suggested that stronger self-resemblance preference for women's faces when progesterone is high could function to increase bonding with female kin when raised progesterone prepares the body for pregnancy and support from kin may be particularly beneficial. However, in the current study, we also found that self-resemblance preference, in comparison with control preference, was weaker when estradiol was higher, but this effect was driven by changes in preferences for control faces. Both estradiol and progesterone are elevated during pregnancy (Johnson, 2007). Thus, these results do not support DeBruine et al.'s (2005) proposal that stronger self-resemblance bias when progesterone is high reflects hormonal regulation of responses to kinship cues that evolved to increase bonding with kin during pregnancy.

One limitation of Study 1 involves measurement. Firstly, some have questioned whether facial resemblance is a valid cue of relatedness (e.g., Giang et al., 2012) and it is certainly not as strong a cue as co-residence for relationships like full siblings. Additionally, the computer graphic manipulation used to create self-resembling faces may not completely capture the cues that indicate kinship. Thirdly, the task of choosing which face looks more attractive or trustworthy in a pair has little ecological validity. Finally, the exact mechanisms controlling potential ovulatory effects are not fully understood. We measured estradiol and progesterone weekly, but measuring E-to-P ratio on this timescale might not be a sensitive enough proxy for the underlying physiological mechanisms, if they exist. While our study *alone* does not provide strong evidence against the idea that women may have some behaviours that function to avoid inbreeding at particularly fertile points across the menstrual cycle, they do provide a clear counterpoint to the interpretation from DeBruine et al. (2005), and contribute to the body of evidence for assessing this hypothesis.

In Study 2, we investigated whether women reported seeking less contact with family and met fewer male kin during their fertile phase. According to our preregistered criterion, we found no evidence for either prediction. However, we found small increases in contact with related men during women's premenstrual phase and menstruation for women using hormonal contraceptives as well as not using hormonal contraceptives. Women also reported seeking more contact with family during menses. Fertile window effects did not reverse or meet our criterion when (pre-)menstruation was not adjusted for. Our sample size for analyses focusing on the number of male kin was limited by design decisions. As a consequence of the small sample size, the low outcome variability, and the large heterogeneity in fertile phase effects across women, credible intervals for the effect of interest were very wide and consistent with effects in both the predicted direction and its opposite. However, our sample size still exceeded that in the previous literature. We also argue that our outcome measure is a more straightforward and ecologically valid operationalisation of the prediction that women will avoid incest than the number of phone calls to fathers used in Lieberman et al. (2011). The high uncertainty in the estimates we report is in no small part due to our choice to model the fertile phase effect as varying between participants. Older work did not always let this effect vary (or at least did not report doing so, as in Lieberman et al., 2011). We believe slopes must be allowed to vary given that we know the fertile phase predictor will approximate the true fertile window more closely for some participants than others. Nevertheless, given the aforementioned drawbacks, the right conclusion is to remain uncertain about this specific effect in view of the evidence we can marshal.

However, we also observed no changes in contact sought with family, where our available sample size was far larger, the item had more variance and credible intervals were, as a result, more narrow. If contact with female kin increases while contact with male kin decreases during the fertile window (as reported by Lieberman et al., 2011), we might expect to see no association on average. However, we believe separate meetings with only mothers or only fathers are rarer than separate phone calls. The inbreeding avoidance hypothesis itself implies no uptick in contact with female kin, but Lieberman et al. (2011) invoked an auxiliary hypothesis to explain increased calls to mothers (increased need to discuss relationship-relevant events). Descriptively, our participants reported a substantial decrease, not increase, in contact with related women in their fertile phase (the 99% CI still included zero). Given that we do not see the countervailing effect reported in Lieberman et al. (2011) in our measure, we argue that the lack of an effect on the time spent with family item is indeed relevant to the incest avoidance hypothesis, though in no way conclusive.

In summary, Study 1 suggests that the results reported by DeBruine et al. (2005) do not reflect mechanisms to avoid inbreeding, while Study 2 suggests that the results reported by Lieberman et al. (2011) may not be robust. In contrast to DeBruine et al. (2005), Study 1 employed a longitudinal design and hormonal measures of fertility, while in contrast to Lieberman et al. (2011), Study 2 included more women on more measurement occasions, a quasi-control group of women using hormonal contraception and a continuous estimate of fertility.

Although Study 2 relied on self-reports, external validity can be considered to be high. Actual contact with related men reflects real world behaviour which should be affected by inbreeding avoidance. Further, this variable should not be strongly affected by potential recall biases. However, for both studies we cannot rule out that our results are sample specific. Despite the aim of Study 2 to recruit a representative sample of women, a majority of our sample were students and resided in a rich, Western country (Henrich et al., 2010), where inbreeding is comparatively rare and heavily proscribed legally and socially.

Zietsch et al. (2015) questioned the overall influence of contextual factors like the ovulatory cycle on variation in preferences. For facial masculinity, genetic variation explained the lion's share of differences in preferences. Recent findings from preregistered studies also hint at the limited influence of the ovulatory cycle on preference shifts (Stern et al. 2020, 2021). However, as these studies still only examined variation within rich, Western countries, the generalisability of the conclusions remains limited. Further replication in different social contexts would be desirable.

In conclusion, combining previous findings with the studies reported here, we remain uncertain whether women's preference for associating with male kin is down-regulated during the ovulatory phase of the menstrual cycle to reduce inbreeding.

## Research transparency and reproducibility

Data files and analysis scripts for Study 1 are publicly available at https://osf.io/wnhma/. Preregistration, data documentation as well as analysis scripts for Study 2 are publicly available at https://osf.io/d3avf/.

## Supplementary material

For supplementary material accompanying this paper visit https://doi.org/10.1017/ehs.2022.41. click here to view supplementary material
