# Supplementary material for: No increased inbreeding avoidance during the ovulatory phase of the menstrual cycle
Source: Evol Hum Sci. 2022 Sep 28;4:e47. doi: 10.1017/ehs.2022.41 (PMC10426077; doi:10.1017/ehs.2022.41)
Supplement: Supplementary file 1 [file S2513843X2200041Xsup001.docx]

# Supplemental Material

**S1. Deviations from Preregistration**

Table S1. Deviations from our preregistration (Study 2)

| Preregistered Method | Deviation | Explanation |
| --- | --- | --- |
| Our preregistered model formula included hormonal contraception only as a moderator of the fertile window effect, not of the (pre-)menstruation variables. | In our main models, we added interaction controls, i.e., interactions between hormonal contraception and (pre-)menstruation. | We now understand that not including interaction controls would lead to an overestimation of the fertile window effect. However, we tested the originally specified model and a model that did not adjust for (pre-)menstruation at all in our robustness checks. Conclusions were unaffected. |
| Our preregistered inclusion criteria were to recruit participants who:   - who report their gender as female (not “other”), - who are younger than 50, - who deem themselves more hetero­ than homosexual, - who are not pregnant or breastfeeding now or during the last three months, - who are not actively trying to become pregnant, - who are not using hormonal contraception or medication now or in the last three months, - who are not using psychopharmacological medication - who deem themselves pre­menopausal - and who report regularly menstruating at the moment. | We did not exclude women:   - who are not actively trying to become pregnant - who are not using psychopharmacological medication (2) - who are not using hormonal medication now or in the last three months (2)   Additional inclusion criteria were:   - being single (1) - not living with parents (1) | We were not sufficiently precise about how we would exclude participants (whether we would do so for main analyses or only in robustness checks). We decided to use one set of criteria for the main analyses but reported robustness analyses with both stricter and less strict criteria. Different exclusion criteria did not change our results.  The two additional inclusion criteria follow from the rationale of our research question. |
|  |  |  |
| Analysis |  |  |
| We preregistered to estimate conception risk based on “whatever is state­-of-­the-­art in the field” (p. 5) and noted that in previous work we used backward and forward counting. | We estimated the probability of being in the fertile window based on backward counting (see Gangestad et al., 2016; Arslan et al., 2018; Stirnemann et al., 2013). As robustness analyses, we reported backward counting from the inferred next menstrual onset, forward counting, backward counting with a squished follicular phase, and a windowed predictor. | At the time of preregistration, we were hoping for further work to be published with superior methods to estimate conception risk. We decided not to specify an exact procedure in order to profit from new insights gained by the time of our analyses. No further algorithms were published to our knowledge, but our own (unpublished) validation work confirms that backward counting is more valid than forward counting (both for predicting conception risk and steroid hormones), that squishing the follicular phase has no discernible additional benefit. Furthermore, we were able to count backwards for most women, so there was less of a tradeoff with sample size than in Arslan et al. (2018). In either case, robustness analyses showed similar results for alternative predictors. |
| Robustness analyses |  |  |
| 1. We preregistered robustness analyses on: 2. whether the results differ by contraceptive method, specifically by whether women are fertility ­aware (i.e. using a counting or temperature method or using a cycle tracking app) 3. whether results are specific to the outcome of interest or driven by more general changes (e.g. whether sexual desire increases go above and beyond any increases in self ­esteem) 4. whether the outcome visually peaks at the estimated day of ovulation when using a generalized additive model or a simpler model across days on the X axis 5. whether excluding various participants who are potentially less likely to ovulate affects the effect size estimate 6. whether the specification of the predictor matters (we will at least compare forward­ vs. backward­ counting, continuous predictor versus window estimation) 7. whether not adjusting for menstruation matters (we predict that it does for some outcomes, e.g., in­pair sexual desire and sexual activity, self ­perceived desirability) 8. whether effect sizes are moderated by    1. age    2. weekday    3. self ­reported average cycle length    4. self ­reported cycle regularity    5. self­ reported certainty about the details of own menstrual cycle    6. self­ reported health | We did not run robustness analysis b) | The robustness analysis specified in b) refers to our main research question and does not provide a reasonable robustness check for our current analyses. In addition, we found no credible main effects.  For some of the planned moderator robustness checks, group sizes for some subgroups were very small. Here, we instead report analyses with these groups excluded (rather than a proper moderation). |

**S2. Correlation between Outcome Variables in Study 2**

Supplementary Note

We were interested whether the "time family" Likert item could serve as workable proxy variable for time spent with male kin, given that our social network variable "reported # male kin" did not work as intended and turned out not to be quite coarse. If we see aggregate time spent with male kin as the criterion, the coarseness of the "# male kin seen for >1h" variable can be seen as criterion invalidity. Criterion invalidity would depress the correlation between *time family* and *# male kin seen*, but it is not relevant for the validity of the time family item for the (unobserved) aggregate time spent with male kin. To understand how valid our time family Likert item is for the latent/unobserved time spent with male kin, we need to look beyond the 0.2 association. Because a disattenuation for reliability is not feasible in this special case, we instead conducted a forward simulation in R to reproduce the observable parameters. We then computed the unobservable correlation between the time spent item and "time spent with male kin". In this simulation, the unobservable correlation turned out to be .49. Of course, given the large number of necessary assumptions, this number should not be taken too seriously. However, it can support the following intuitions: **a.** given the coarseness of the *# male/female kin* variable, their .44 correlation is an indication that participants frequently saw both male and female relatives on one day. **b.** even when making conservative assumptions (such as the Likert item being affected by more measurement/reporting error than social network listings), there is room for a substantial correlation between the Likert item and the unobserved time spent with male kin, even while the correlation with the reported number of male kin is low.

The simulation made the following assumptions:

- We simulated time spent with both parents as having an average of 18 minutes per day.
- Parents were usually seen together, but were seen on their own for 3/11 minutes per day on average (for fathers/mothers).
- We simulated two further male and female kin who were seen on 3 minutes per day on average.
- We simulated a measurement/memory error of 12 minutes for every time-based measure (time spent with each male/female kin). We assumed double the amount of error for the Likert item, and we assumed that memories of meetings with parents had perfectly correlated measurement error.
- We then computed the number of male/female kin seen for more than an hour per day from the minute variable for each relative. We did not simulate the flaws in our procedure, such as the fact that not all social network members were rated.
- We verified that this procedure approximately reproduced the observable mean for # male kin seen for >1h (0.056).
- We verified that this procedure approximately reproduced the observable mean for # female kin seen for >1h (0.095).
- We verified that this procedure approximately reproduced the observable correlation between # male/female kin (r = 0.44).
- We simulated a response to our 5-point Likert item by adding measurement error, square-root-transforming the number of minutes with family and then pressing the data into the bounded scale, so that 0 reflected no time with family, and 4 the maximal time with family.
- We verified that this procedure approximately reproduced the observable correlation between # male kin seen >1h and the time family item (r = 0.24).
- We computed the correlation between the simulated time family item and the square root of the latent/unobserved number of minutes spent with male kin (r = 0.49).
- We also computed the correlation between the simulated # male kin and and the square root of the unobserved number of minutes spent with male kin (r = 0.49).

**S3**. **Further Analyses**

**Figure S1.** Change in number of male kin seen over the menstrual cycle in the subset of women who showed intraindividual variation in the outcome.
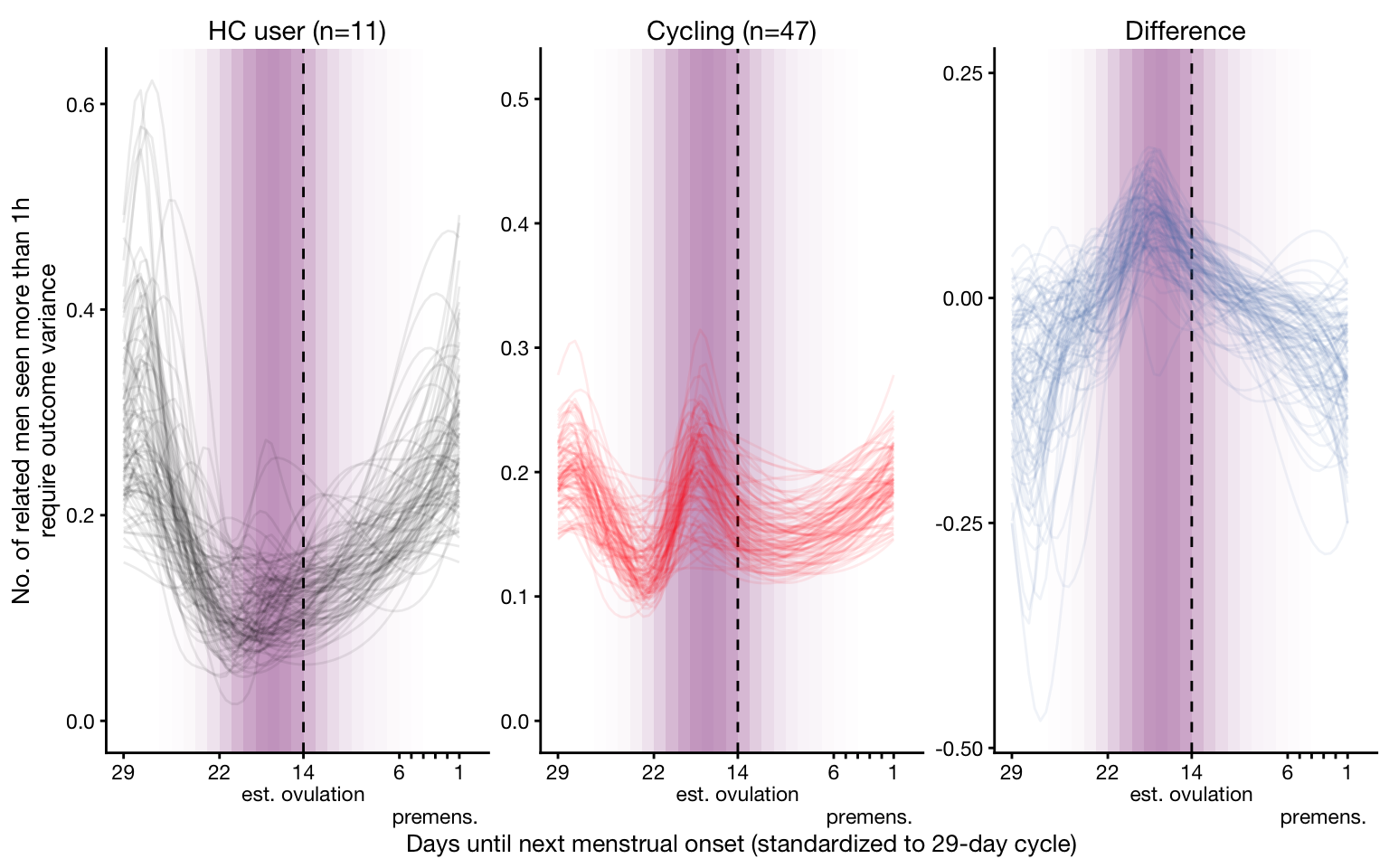


**Table S2.** Model summary of the specificity models.

|  | **# unrelated men** | | **# female kin** | | **# unrelated women** | |
| --- | --- | --- | --- | --- | --- | --- |
| *Predictors* | IRRs | 99% CIs | IRRs | 99% CIs | IRRs | 99% CIs |
| Intercept | 0.20 | 0.13; 0.27 | 0.02 | 0.01; 0.03 | 0.21 | 0.14; 0.29 |
| Premenstrual phase | 0.93 | 0.82; 1.05 | 0.99 | 0.82; 1.19 | 0.94 | 0.85; 1.03 |
| Menstruation | 1.07 | 0.93; 1.21 | 0.96 | 0.77; 1.21 | 0.99 | 0.88; 1.11 |
| Fertile phase | 0.97 | 0.66; 1.37 | 0.39 | 0.06; 1.50 | 0.89 | 0.60; 1.29 |
| Hormonal Contraception (HC) | 0.87 | 0.39; 1.66 | 1.04 | 0.22; 3.73 | 0.83 | 0.36; 1.64 |
| Premenstrual phase ✕ HC | 0.97 | 0.73; 1.27 | 1.07 | 0.64; 1.77 | 1.13 | 0.88; 1.47 |
| Menstruation ✕ HC | 0.96 | 0.68; 1.30 | 1.53 | 0.85; 2.70 | **1.36** | **1.04; 1.79** |
| Fertile phase ✕ HC | 1.20 | 0.67; 2.08 | 1.73 | 0.31; 7.15 | 1.27 | 0.69; 2.24 |

*Note.* Group-varying effects omitted, see online documentation.

**S4. Robustness Checks**

**Figure S2.** Robustness checks for the number of male kin outcome.


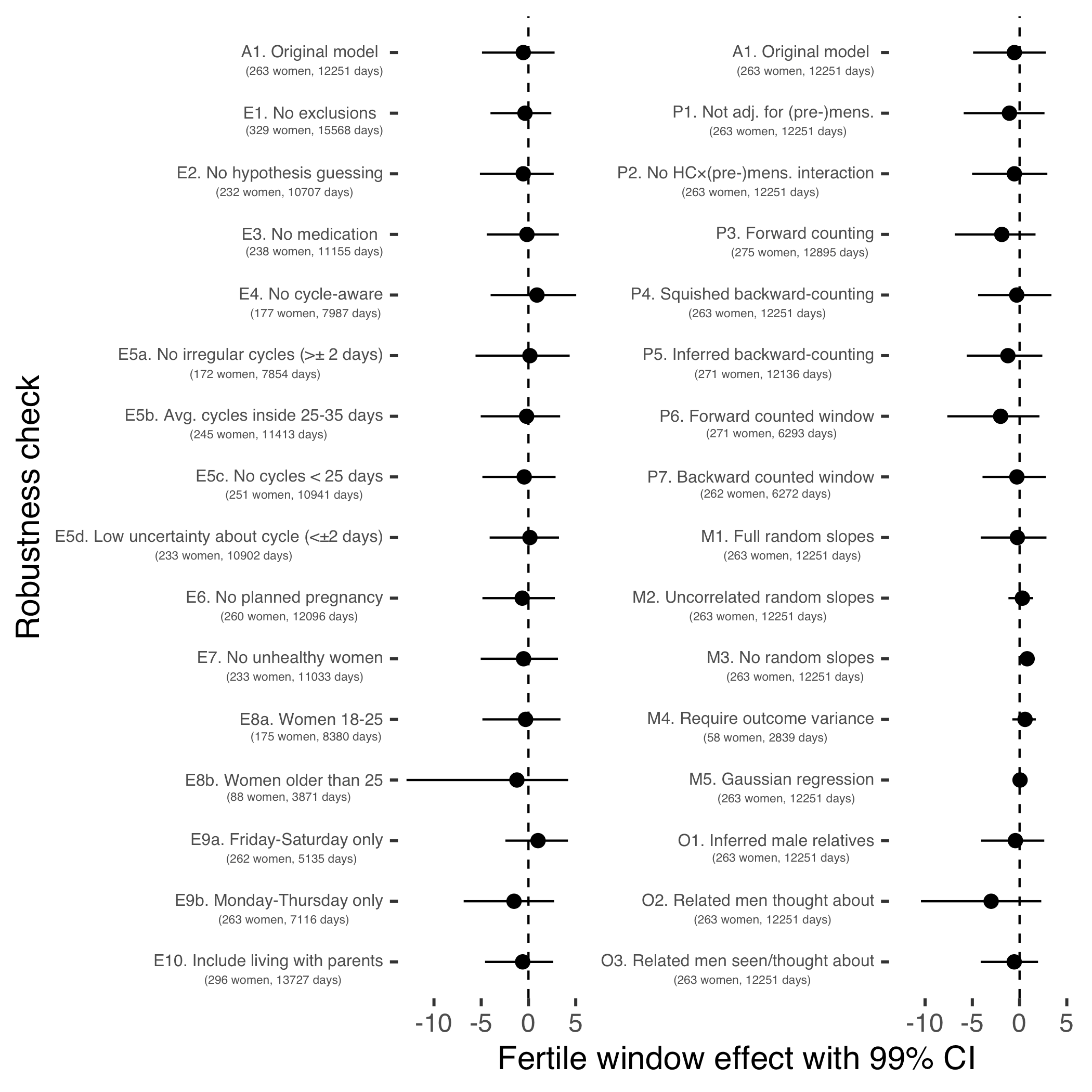


Notably, when we omitted varying slopes (M2), as in Lieberman et al. (2011), the association was positive in the opposite direction of the prediction.
